# Metabolic syndrome among individuals living with hypertension in Accra, Ghana

**DOI:** 10.1371/journal.pone.0253837

**Published:** 2021-10-20

**Authors:** Aaron Kobina Christian, Olutobi Adekunle Sanuade, Sandra Boatemaa Kushitor, Mawuli Kushitor, Irene Kretchy, Charles Agyemang, Ama de-Graft Aikins

**Affiliations:** 1 Regional Institute for Population Studies (RIPS), University of Ghana, Legon-Accra, Ghana; 2 Center for Global Cardiovascular Health, Northwestern University Feinberg School of Medicine, Chicago, Illinois State, United States of America; 3 Food Security Initiative and Centre for Complex Systems in Transition, Stellenbosch University, Stellenbosch, South Africa; 4 Department of Health Policy, Planning and Management, University of Health and Allied Sciences, Ho, Ghana; 5 Department of Pharmacy Practice and Clinical Pharmacy, School of Pharmacy, University of Ghana, Accra, Legon-Ghana; 6 Department of Public Health, Amsterdam UMC, University of Amsterdam, Amsterdam Public Health Research Institute, Amsterdam, The Netherlands; 7 Institute of Advanced Studies, University College London (UCL), London, United Kingdom; Makerere University School of Public Health, UGANDA

## Abstract

Metabolic syndrome (MetS) is a major risk factor to cardiovascular diseases. In this study, we investigate the prevalence and associated risky behaviour of MetS in resource-poor urban communities in Accra, Ghana. We analysed data on 111 persons with hypertension, screened and recruited for a therapeutic lifestyle intervention program in August 2015. MetS was measured using the International Diabetes Federation (IDF) and the World Health Organization definitions. The prevalence of MetS was 58.4% and 16.8% by the IDF and WHO definitions respectively. More women (61.8%) compared to men (31.8%) had MetS (*p* = 0.011). Approximately 31% of the hypertensive patients were engaged in moderate-intensity physical activity; 9.0% were current smokers, 42.0% consumed excess alcohol over the past month prior to the interview and 41.0% discontinued taking their antihypertensive medications without consulting with a doctor. About 42.0% and 37.0% of respondents always consumed fruits and vegetables respectively at least two times a day. The binary logistic regression showed that compared to women, men had lower odds of consuming two or more servings of vegetable in a day (OR: 0.2; 95% CI; 0.1, 0.8). Increase in age was associated with higher odds of consuming fruits at least twice a day (1.0; 1.0, 1.1) but with lower odds of engaging in moderate intensity physical activity (0.9; 0.8, 1.0). Being married was associated with higher odds of engaging in moderate physical activity (2.8; 1.0, 8.2). Therapeutic methods essential for the management of patients with hypertension and MetS should include non-pharmacological remedies targeting the promotion of medication adherence, Dietary Approaches to Stop Hypertension (DASH) and physical activities; these are vital to changing unhealthy lifestyle which worsens the underlying pathology.

## Background

Metabolic Syndrome (MetS) is a condition characterized by a group of risk factors resulting from the malfunction of the body’s biomedical processes which increases the risk of cardiovascular diseases (CVD) and complicates the management of other chronic non-communicable diseases (NCDs) [1–9]. Although the critical contribution of NCDs to the region’s disease burden is often overlooked [10], both projected and current age specific mortality rates from NCDs, particularly among young adults in this region are higher compared to that of most high-income countries [11–16]. The current nutrition transition observed in many urban spaces in developing countries suggests an increase in some western dietary habits including fast foods which is high in carbohydrate and salt, moderate protein and has average fiber content [17]. Some observable lifestyle modifications such as lack of adequate physical activities, poor eating habits, high alcohol consumption, smoking and substance abuse contribute significantly to the current disease landscape [18–23]. These setting provides favorable conditions for MetS and major risk factor for hypertension and other NCDs.

NCDs are now major causes of disability and deaths in Ghana. The burden of risk factors leading to various NCDs in Ghana has been underestimated [24]. The World Health Organization report on NCDs country profiles in 2018 reveals that NCDs caused the death of estimated 94,400 persons in Ghana in 2016 [25] with hypertension and cardiovascular diseases (CVDs) do constituting a significant proportion of the country’s NCD burden. Compared to HIV with a prevalence of 1.8%, the prevalence of hypertension in rural and urban centres ranges from 27.0% to 32.3% respectively [26]. Hypertension currently ranks fifth in outpatient mortality nationwide but second in the Greater Accra Region [27].

Diagnosing MetS is meant to identify persons with an elevated risk of both type 2 diabetes and cardiovascular diseases [28]. Although rise in hypertension could exacerbate the burden of MetS and vice versa, there is limited information on the burden of MetS among people living with hypertension from community surveys in developing countries such as Ghana. The purposes of the present study were 1) to investigate the prevalence of metabolic syndrome among hypertensives living in resource poor urban communities in Accra, Ghana using the IDF and WHO definitions of the condition 2) and examine individual risky behaviour of MetS

## Data and methods

### Study design

This was a cross-sectional study. Analysis was based on the baseline data of a community-based psychosocial intervention addressing cardiovascular diseases [29]. The project was titled “Developing community-based cardiovascular disease care in Ghana: a therapeutic lifestyle approach to hypertension management in Ga Mashie, Accra”- “Tsui Anaa”.

### Study area

This study was carried out in two urban poor communities, James Town and Ussher Town, in Accra, Ghana. With funding from the Secretariat of the African Caribbean and Pacific Group of States–ACP-EU Cooperation Programme in Higher Education (EDULINK) and IDRC, the Regional Institute for Population Studies, University of Ghana, established a demography and population health research field site in the two communities, typically known as Ga Mashie. Some of the oldest settlements in Accra are located in Ga Mashie and the major livelihoods of community members include trading, fishing and fishing related activities. The two communities are densely populated, with poor access to social amenities. Further details about the study sites have been provided elsewhere [27, 30–33].

### Sampling design and sample

The sampling frame for the EDULINK research involves the systematic selection of forty households from 29 randomly selected enumeration areas. After three waves of data collection conducted at approximately 18-month intervals in 2010 (June), 2011 (December) and 2013 (August) with households and individuals, 202 persons self-reported as having been diagnosed hypertension by medical personnel. These individuals were invited to take part in the Tsui Anaa study, however, only 111 participated.

### Data collection

Baseline data of individuals with elevated diastolic and systolic blood pressure (individuals with hypertension) who agreed to be part of the intervention program were collected in August 2015. Questionnaires used were guided by previous experience grain from surveys conducted in the study area over the past decade. All questionnaire used were pretested and reviewed before administering to study respondents. Data collected by trained and experienced enumerators included: a) sociodemographic characteristics (age, marital status, education, occupation, and ethnicity), lifestyle habits (smoking and alcohol consumption status) and current medications use; b) anthropometric measurements: Body weight and height, measured to the nearest 0.1 kg and centimetres respectively using a digital scale and a carpenter’s tape measure). Waist and hip circumference were measured with a flexible and non-distensible tape; All anthropometric measurement were done by two trained examiners. Two examiners were used for logistical reason and also because it was the minimum required to check inter examiner reliability. Collections of biochemical assays which included the collection of venous blood by a trained biomedical officer after a 12-h overnight fast; serum samples were evaluated for total cholesterol, HDL cholesterol, and triglycerides using standard methods at the Noguchi Memorial Institute for Medical Research at the University of Ghana, Legon; d) Blood pressure measurements taken with a standard mercury sphygmomanometer following a recommendation by the America Heart Association by trained health personnel’s [34, 35]. Respondents blood pressure readings were measured using the Omron Automatic Upper Digital Blood pressure monitor (HEM-907) at intervals of 10 minutes or more.

### Ethics statement

The study was approved by the Institutional Review Board of the Noguchi Memorial Institute for Medical Research at the University of Ghana, Legon. The ethical clearance number was 088/13-14. All the participants provided written informed consent.

### Measures

#### Metabolic syndrome

MetS was estimated using the International Diabetes Federation (IDF) and World Health Organization (WHO) definitions [2, 28, 36]. Given that respondent in this study were all individuals living with hypertension, MetS definition given by the IDF is as follows; Waist Circumference ≥ 94cm in men or ≥80cm in women plus one or more of the following:

- Low High -density lipoprotein (HDL): (<40mg/dl in males or <50mg/dl in females) or specific treatment for this lipid abnormality.
- Hypertriglyceridemia (TG≥150mg/dl) or specific treatment for this lipid abnormality.
- Dysglycaemia (Fasting Plasma Glucose (FPG) ≥100mg/dl) or previous diagnosis of type 2 diabetes [2].

The MetS definition by WHO is as follows [37]; hyperinsulinemia or hyperglycemia (fasting Plasma glucose ≥ 110 mg/dl) in addition to one or more of the following:

- Hypertriglyceridemia (TG≥150mg/dl i.e., ≥ 1.7 mmol/l) or specific treatment for this lipid abnormality
- Low High-density lipoprotein (HDL): (< 40 mg/dl or 1.03 mmol/l in males or < 50mg/dl i.e., 1.29 mmol in females)
- BMI: > 30 kg/m^2^

#### Excessive alcohol intake

Female respondents who reported consuming four or more drinks of the same alcohol beverage on a single occasion and male respondents who reported consuming five or more drinks of the same beverage type on a single occasion [38].

#### Vegetable and fruits consumption

Respondent who reported consuming at least two servings of fruits and/or vegetables a day were considered as having a positive dietary practice with respect to fruits and vegetables. Examples of commonly consumed fruits in study area are oranges, bananas, pineapples and mangoes.

#### Dietary management knowledge

Recommended dietary management of NCDs includes reduction of refined sugars and fat intake and the increase consumption of dietary fiber. Respondents who responded to having knowledge about how to reduce dietary sugar and fat and increase dietary fiber were considered to have appropriate dietary management knowledge with respect to NCDs.

#### Moderate-intensity physical activity

Respondents were asked about the number of days per week for which they engage in activities that cause small increase in breathing or heart rate such as brisk walking (or carrying light loads) for at least 10 minutes continuously. Those who engaged in such activities for 1–4 times over the past seven days were termed as engaging in moderate-intensity physical activity [31].

#### Medication adherence

Respondent were asked if they were taking any prescribed orthodox medications for the management of hypertension.

### Data analysis

The data were analysed using Stata version 12 [39]. Continuous clinical and biochemical data of study subjects were expressed as means and standard deviations and proportions were used to represent discrete/categorical variables. Continuous data were compared using unpaired t-test. Chi-square tests were used for differences in the proportion of categorical variables between groups.

Given the critical role central obesity (i.e., elevated waist circumference) is in predicting chronic diseases such as hypertension and diabetes particularly in sub-Saharan populations [40], further analysis to determine of how MetS influences lifestyle behavior was conducted with the IDF criteria which considers central obesity as a mandatory requirement to be diagnosed as having MetS unlike the WHO definition. Additionally, the use of the IDF definition of MetS is more suited in used more in community surveys such as current study as oppose to the WHO criteria for MetS [41, 42]. A binary logistic regression analysis was conducted to determine selected sociodemographic correlates of specific lifestyle behaviour linked to either risk or management of NCDs.

## Results

Table 1 shows the background characteristics of the study respondents. The study sample had more females (n = 89) than males (n = 22). The mean age of respondents was 57 ± 11.2 with no statistical difference between the ages of the males and females. More than half (51.4%) were not married, and the proportion was significantly higher among females than males. The predominant ethnic group was the Ga-Damgbe. Compared to women in the study group, significantly more males were married and belonged to the Ga-Damgbe ethnic group. A little over a tenth (10.8%) of respondents had no formal education and approximately 21% had secondary education or more.

**Table 1:** Selected background characteristics, anthropometric indices and biochemical assays of study participants by sex.

Characteristics	Total (N = 111)	Female (n = 89)	Male (n = 22)	P-Value
**Marital Status**				
Not Married	51.4 (57)	56.2 (50)	18.2 (4)	0.001^a^
Married	48.6 (54)	43.8 (39)	81.8 (18)	
**Educational Status**				
No Education	10.8 (12)	11.2 (10)	9.1 (2)	0.312^a^
Primary	32.4 (36)	36.0 (32)	18.2 (4)	
Middle/JHS	36.0 (40)	34.8 (31)	40.9 (9)	
Secondary +	20.7 (23)	18.0 (16)	31.8 (7)	
**Ethnicity**				
Ga-Damgbe	82.0 (91)	87.6 (78)	59.1 (13)	0.002
Other	18.0 (20)	12.4 (11)	40.9 (9)	
**Age**	57.0 ± 11.2	57.5 ± 11.8	58.7 ± 8.9	0.130
**Anthropometric indictor**				
Waist circumference (n = 104)	100.3 ± 23.0	100.3 ± 24.5	100.4 ± 16.5	0.041
BMI (Kgm^-2^)	32.3 ± 8.1	32.9 ± 8.1	29.8 ± 7.7	0.792
Normal	16.4 (17)	13.3 (11)	28.6 (6)	0.234
Overweight	31.7 (33)	32.5 (27)	28.6 (6)	
Obese	51.9 (54)	54.2 (45)	42.9 (9)	
**Biochemical Assays**				
TG (mmol/l)	7.3 ± 3.4	7.5 ± 3.2	6.8 ± 3.8	0.315
Normal	64.9 (72)	61.8 (55)	77.3 (17)	0.173
Dyslipidaemia	35.1 (39)	38.2 (34)	22.7 (5)	
HDL-C(mmol/l)	1.2 ± 0.3	1.2 ± 0.3	1.1 ± 0.2	0.015
Normal	45.9 (51)	40.4 (36)	68.2 (15)	0.019
Low HDL-C	54.1 (60)	59.6 (53)	31.8 (7)	
FBG (mmol/l)	5.3 ± 2.9	5.4 ± 3.0	4.9 ± 2.5	0.257
Normal	67.6 (75)	63.3 (59)	72.7 (6)	0.564
Diabetic	32.4	33.7 (30)	27.3 (6)	
**Hemodynamic Parameters**				
SBP (mmHg)	151.2 ± 25.1	150.0 ± 25.3	155 ± 24.4	0.330
DBP (mmHg)	96.3 ± 14.8	95.2 ± 14.9	100.5 ± 14.1	0.135
Duration of hypertension in months	85.4 ± 87.3	83.5 ± 84.3	87.4 ± 90.0	0.661
**Metabolic Syndrome**				
IDF MetS Criteria	58.4 (59)	64.2 (52)	35.0 (7)	0.018
WHO MetS Criteria	16.8 (17)	17.3 (14)	15.0 (3)	0.554*

Values presents mean ±standard deviation (SD) or % (n). Continuous data were compared using unpaired t-test whilst categorical data were compared using chi-square and Fischer’s exact test (^a^) where indicated.

Except for the mean value for fasting blood glucose (FGP), the mean values for waist circumference, BMI, triglycerides, HDL-cholesterol, SBP and DBP were all above normal recommended levels. The mean waist circumference of men was significantly higher than that of the women (p = 0.041), while the mean HDL-cholesterol of the women was greater than the men (P = 0.015).

The prevalence of obesity, diabetes, and dyslipidaemia (elevated triglycerides) and low HDL was 48.6%, 32.4%, 35.1% and 54.1% respectively among the participants. Significantly, a higher proportion of males (60.0%) had low HDL compared to the females (31.8%).

The prevalence of MetS was 58.4% and 16.8% using the IDF and WHO criteria respectively. Significantly higher proportions of females (64.2%) compared to males (35.0%) had MetS using the IDF criterion.

### Risk behaviours for metabolic syndrome or NCDs

Approximately 36.6% and 42.6% of the respondents consumed at least two servings of vegetables and fruits a day either frequently or often, respectively (Table 2). A little above a third (32.7%) were excessive alcohol consumers.

**Table 2:** Selected lifestyle behaviour characteristics by respondents’ metabolic syndromes status.

Lifestyle behaviour	Total	MetS status		*p*-value
		Non-MetS	MetS	
		N = 42	N = 59	
Vegetables				
Sometimes consumes two serving per day	63.4 (64)	69.1 (29)	59.3 (35)	0.317
Always/often eats at least two servings per day	36.6 (37)	30.9 (13)	40.7 (37)	
Fruits				
Sometimes consumes two serving per day	57.4 (58)	61.9 (26)	54.2 (32)	0.442
Always/often eats at least two servings everyday	42.6 (43)	38.1 (16)	45.8 (43)	
Alcohol consumption				
Excessive alcohol	32.7 (33)	38.1 (16)	28.8 (17)	0.327
Smoking				
Currently smoking	9.9 (10)	16.7 (7)	5.1 (3)	0.055
Physical activity				
Moderate intensity	32.7 (33)	23.8 (10)	39.0 (23)	0.109
Knowledge of risk reduction				
Reduction of fats	63.4 (64)	59.5 (25)	66.1 (39)	0.499
Reduction of sugars	63.4 (64)	57.1 (24)	67.8 (42)	0.273
Increase dietary fibre	49.5 (50)	38.0 (21)	57.6 (34)	0.053
Medication adherence				
Stop taking medications when symptoms are under control	42.6 (43)	45.2 (19)	40.7 (24)	0.648

Values presented as percentages with frequencies in parenthesis. Comparison determined using chi-square test.

Of all risk behaviours reported, smoking featured the least (9.9%) among the current study sample. Approximately 50.0% and 63.4% of the participants had some knowledge about how to increase their dietary fibre intake and reduce their dietary sugar respectively. For the control of fat and cholesterol, approximately 63.4% indicated they had some knowledge of appropriate meals or meal regimes to consume. Significantly, more respondent living with MetS had some knowledge about how to increase dietary fibre than their counterpart who did not have MetS

Further, about a-third of the respondents (32.7%) were engaged in moderate-intensity physical activity. Over two-fifth (42.6%) reported to cut back or stop taking medication without informing a doctor anytime they felt their condition had improved. Except for currently smoking, there were no significant differences between the proportion of individuals with or without Mets with respect to selected lifestyle behaviour.

### Correlates of lifestyle behaviour

Table 3 shows the relationship between MetS status and lifestyle behaviour of study respondents, controlling for selected sociodemographic factors. The results showed that MetS status only was associated with knowledge of dietary management, specifically the increase of dietary fiber in meals. The odds of having knowledge concerning dietary risk management were higher for those with MetS compared to those without MetS [Odds Ratio = 3.4, 95% CI: 1.3–8.6]. Sex was the only factor associated with vegetable consumption, and the results showed that males were less likely to consume at least two servings of vegetables daily compared to their female counterparts. With respect to fruit consumption, advancement in age significantly increased the odds of consuming at least two (2) servings of fruits daily. The odds of consuming excess alcohol were higher for those living in Ussher Town compared with their counterparts in James Town.

**Table 3:** Correlates of lifestyle behaviour associated with the risk of NCDs.

	Vegetable consumption^1^	Fruit Consumption^2^	Moderate intensity Physical Activity	Alcohol Consumption^3^	knowledge of dietary risk management-Increase fiber intake^4^
Variable	OR [95% CI]	OR [95% CI]	OR [95% CI]	OR [95% CI]	OR [95% CI]
MetS (ref: Non-MetS)					
MetS	1.2 [0.5, 3.1]	1.3 [0.5, 3.4]	1.6 [0.6, 4.7]	0.83 [0.33, 2.0]	3.4 [1.3, 8.6]**
Sex (ref: Female)					
Male	0.2 [0.1, 0.8]**	0.6 [0.2, 1.9]	0.6 [0.2, 2.2]	2.21 [0.71, 6.8]	2.4 [0.7, 7.6]
Marital Status (ref: Single)					
Married	1.1 [0.4, 3.1]	1.1 [0.4, 3.0]	2.8 [1.0, 8.2]*	0.97 [0.93, 1.0]	0.5 [0.2, 1.3]
Formal education					
Primary	2.8 [0.6, 14.4]	4.3 [0.8, 22.4]*	0.4 [0.1, 2.4]	0.7 [0.2, 3.7]	0.8 [0.2, 3.6]
Middle/JHS	1.9 [0.4, 9.3]	3.7 [0.7, 18.9]	0.7 [0.1, 3.6]	0.4 [0.1, 1.9]	0.6 [0.1, 2.8]
Secondary+	2.4 [0.4, 13.6]	4.1 [0.7, 23.2]	0.5 [0.1, 3.0]	0.6 [0.1, 3.4]	1.2 [0.3, 6.2]
Age in years	1.0 [1.0, 1.1]	1.0 [1.0, 1.1]**	0.9 [0.9, 1.0]**	0.9 [0.9,1.0]	1.0 [1.0, 1.1]
Place (Ref: James Town)					
Ussher Town	1.7 [0.0, 1.4]	1.54 [0.67, 3.5]	1.1 [0.4, 3.0]	3.5 [1.4, 9.1]**	0.7 [0.0, 4.7]

***P≤0.001

**P≤0.05

*P≤0.1.

^1^Consuming at least two serving of vegetables per day.

^2^ Consuming at least two serving of fruits per day.

^3^ Consuming excess amount of alcohol (5 or more per single occasion for men and 4 or more for women).

^4^Knowledge of dietary risk management of chronic disease (increasing consumption of fiber).

## Discussion

This study shows that more than half of all the individuals with hypertension enrolled for the Tsui Anaa study in Jamestown and Ussher Town had MetS. The prevalence of MetS in this population was higher than the 31.7% and 34.6% prevalence observed among urban Black adult population in both Cape Town, South Africa and in Seychelles respectively [43]. However, this higher prevalence of MetS may be attributed to the fact that all respondents screened for this study were purposively confirmed persons with hypertension. Consistent with other studies, the prevalence of MetS varies among men and women [44–46]. Similarly, higher prevalence of metabolic syndrome among women compared to men have been reported in rural South Africa (women: 25% vs. men 10.5%) and among an urban population in Kenya (women: 40.2% vs. 29%) [47, 48].

The higher prevalence of MetS observed among women may be attributed to the increasing trend of overweight and obesity seen in women in this region. Using data from the Demographic and Health Survey of 24 different countries, Amugsi et al. (2017) reported an increase in overweight and obesity among women of reproductive age particularly in urban Africa, with an observed doubling or tripling of obesity among this age group over approximately a decade [49]. Using a case study of South Africa, some reasons given for the higher obesity rate in women compared to men are that; nutritionally deprived girls were more likely to be obese women, whereas being nutritionally deprived as a boy had no significant effect on the risk of obesity in adulthood [50]. Also, an improvement in socioeconomic status in women was associated with obesity level, an effect absent in men [50]. Contrarily, Peer et al. (2015) observed that this trend of higher prevalence of MetS among women compared to men in most sub-Saharan African countries is not observed in African Americans and in high income countries.

The results showed that knowledge of dietary management was significantly higher among those with MetS. This is not surprising because those with MetS usually receive information and counselling on lifestyle modification from health care professionals [51]. Increased consumption of dietary fiber is recommended as it is considered to modulate parameters associated with metabolic syndrome, such as general food intake, glycemia, insulinemia, blood lipids and blood pressure [52]. Less than half of the respondents, though being individuals living with hypertension were consuming fruits and vegetables always or often at least twice a day. This was particularly worrying given the importance of consuming fruit and vegetables in the dietary management of MeTS and chronic disease in general. Similarly, a using data from the Ghana Demographic and Health Survey (GDHS) Ghose et al (2018) concluded that most women were not consuming the recommended servings for fruits and vegetables [53]. Considering that the consumption of fruits and vegetables is linked to improve the various indicators for MetS, their consumption should be encouraged. For example, in a 12-week, placebo-controlled, randomized intervention, although the consumption of cranberry extracts had no effect on blood pressure it lowered total and LDL cholesterol, and total/HDL-cholesterol ratio [54]. Also, the consumption of strawberry for a 6-week period was found to have significantly lowered diastolic blood pressure, but with no changes in systolic blood pressure as well as serum triglycerides and cholesterol levels [55]. This notwithstanding, the results of this study is consistent with a similar study conducted in China that showed only a-third of respondents consumed adequate amounts of fruits and vegetables [55].

Evidence shows that people who consume 4 or more servings of fruits per day have lower odds of hypertension compared to those who do not [55]. Thus, there is a conclusive evidence that long-term intake and increased consumption of fruits may reduce the risk of developing hypertension [56]. On the other hand, there is inconclusive evidence on vegetable consumption and risk of hypertension, albeit the DASH recommends a 4-to 6 servings of vegetables per day [57]. Comparing the recommended intake of vegetable to the less than 30% of the respondents who consumed at least 2 servings of vegetable per day shows the inadequate consumption of vegetables among the study participants. A meta-analysis based on observational evidence concluded that the consumption of fruits and vegetables was associated with a significant decrease in the risk of MetS [58]. However, adequate dose-response studies which provide consistent information on the constitution of adequate consumption of vegetable are limited.

Given that these were confirmed persons with hypertension, the proportion of respondents consuming excessive alcohol was relatively high. Whereas excessive intake of alcohol increases the risk of MetS, light to moderate intake of alcohol has been shown to have the potential to reduce the risk of hypertension and other NCDs [59]. The beneficial effect of light-moderate consumption of alcohol is however skewed towards the consumption of wines and can partly be linked with the presence of antioxidants (e.g., resveratrol) contained in wine [60]. This assertion is however not supported by other studies. McCann et al 2003 also stated that individuals consuming or preferring moderate amounts of wine over high intake of beer or spirits tend to have favourable sociodemographic and lifestyle behaviour (i.e., higher education and wealth, older age, more female than male, and eat a relatively healthier diets) that decreases their risk of NCDs [61]. Thus, recommendations concerning consumption of light to moderate amount of alcohol as a presentation of hypertension and other NCDs remains highly controversial.

Smoking is considered to play a causal role in the development of MetS because it usually causes a rise in sympathetic activity and increases circulating cortisol, catecholamines, vasopression and growth hormone levels [62, 63]. This current study showed a lower proportion of persons with hypertension smoking (approximately 10.0%) compared to what has been reported among Hispanic, White, and African Americans in the USA [64]. The findings further showed that a significantly higher proportion of individual without MetS were smoking compared to those with metabolic syndrome. This might be as a result of the awareness of multiple metabolic conditions of patients motivating them not to smoke. Evidence from a systematic review of randomized control trails shows that moderate intensity exercise prevents hypertension and/or help in the management of other NCDs including MetS [65]. Hence the fact that only 30 percent of study participants engage in moderate-intensity physical activities needs attention.

More than half of the study participants had some knowledge concerning selected dietary recommendations for the management of hypertension. This shows a clear disconnect between the proportion of respondents with specific knowledge about dietary recommendation and the proportion consuming fruits and vegetables at least 2 times a day. This further buttress the fact that knowledge does not always translate into practice. There is ample evidence suggesting the benefits of dietary management of chronic diseases including metabolic syndrome, however, adherence to recommendations has proved unsuccessful in several studies. For example, a study showed that a higher proportion of hypertensives were reported to be heavy alcohol takers compared to normotensives [66]. High rates of smoking, physical inactivity couple with poor dietary habits have been reported among known hypertensives in several other population-based studies [67, 68].

A study conducted in the same research communities showed that the built environment in these communities can be described as ‘obesogenic [69]. Like most urban poor communities, the options for fruits, vegetables and the availability of physical activities spaces are very limited. On the contrary, there is an abundance of out of home cooked foods and convenience stores selling high fatty and sweeten foods and snacks [27]. Hence, the nature of these communities increases the risk of MetS. Therefore, it is important that appropriate intervention strategies are put in place to address this obesogenic environment.

This study has some limitations. The study population was not representative of the entire population of the two communities, as the selection and recruitment of the respondents into the study were based on non-random sampling (i.e., purposive sampling). Therefore, the findings should be interpreted with caution. This study was also based on small sample size and this limited rigorous analysis. This may explain why some of the risk behaviours were not significantly associated with MetS as other studies have reported. Additionally, given the cross-sectional nature of study, we could only provide evidence of associations and did not make any evidence about causality. Finally, the MetS prevalence reported in this study was not standardized; there is a possibility of over-estimation of the actual prevalence of MetS in these communities.

## Conclusion

Although MetS is not routinely checked in the current health screening in Ghana, this study demonstrates that more than half of individuals with hypertension in resource-poor urban communities in Accra showed evidence of MetS. The study also observed poor dietary and lifestyle behaviours as well poor adherence to prescribed antihypertensive medications which can further lead to metabolic abnormalities. There is, therefore, the need to manage patients with hypertension and metabolic syndrome in poor resource communities with an integrated therapeutic approach with both pharmacological and non-pharmacological interventions targeting the promotion of medication adherence, encourage the DASH concept and engaging in physical activities. There is also a critical need for social and health protection interventions to support the most disadvantaged community members who cannot afford ‘the healthy lifestyles’ required for NCD risk reduction.
